# Positioning of Minimally Invasive Liver Surgery for Hepatocellular Carcinoma: From Laparoscopic to Robot-Assisted Liver Resection

**DOI:** 10.3390/cancers15020488

**Published:** 2023-01-12

**Authors:** Shogo Tanaka, Shoji Kubo, Takeaki Ishizawa

**Affiliations:** Department of Hepato-Biliary-Pancreatic Surgery, Osaka Metropolitan University Graduate School of Medicine, Osaka 545-8585, Japan

**Keywords:** hepatocellular carcinoma, laparoscopic liver resection, long-term outcomes, robot-assisted

## Abstract

**Simple Summary:**

Laparoscopic liver resection is widely accepted in the surgical treatment of hepatocellular carcinoma. Laparoscopic liver resection has been reported to result in earlier postoperative recovery and fewer postoperative complications than open liver resection for hepatocellular carcinoma. Laparoscopic liver resection is technically feasible for selected patients with hepatocellular carcinoma even under several situations such as the prevalence of liver cirrhosis, obesity, elderly, hepatocellular carcinoma recurrence (repeat liver resection), and major resection that led to better intra- and post-operative outcomes than open liver resection. In recent years, robot-assisted liver resection has gradually become popular, and its short- and long-term results for hepatocellular carcinoma are reported to be not different from those of laparoscopic liver resection. Robot-assisted liver resection is expected to become the mainstay of minimally invasive surgery in the future.

**Abstract:**

Laparoscopic liver resection (LLR) is widely accepted in the surgical treatment of hepatocellular carcinoma (HCC) through international consensus conferences and the development of difficulty classifications. LLR has been reported to result in earlier postoperative recovery and fewer postoperative complications than open liver resection (OLR) for HCC. However, the prevalence of liver cirrhosis, obesity, the elderly, HCC recurrence (repeat liver resection), and major resection must be considered for LLR for HCC. Some systematic reviews, meta-analysis studies, and large cohort studies indicated that LLR is technically feasible for selected patients with HCC with these factors that led to less intraoperative blood loss, fewer transfusions and postoperative complication incidences, and shorter hospital stays than OLR. Furthermore, some reported LLR prevents postoperative loss of independence. No difference was reported in long-term outcomes among patients with HCC who underwent LLR and OLR; however, some recent reports indicated better long-term outcomes with LLR. In recent years, robot-assisted liver resection (RALR) has gradually become popular, and its short- and long-term results for HCC are not different from those of LLR. Additionally, RALR is expected to become the mainstay of minimally invasive surgery in the future.

## 1. Introduction

**Figure 1:**
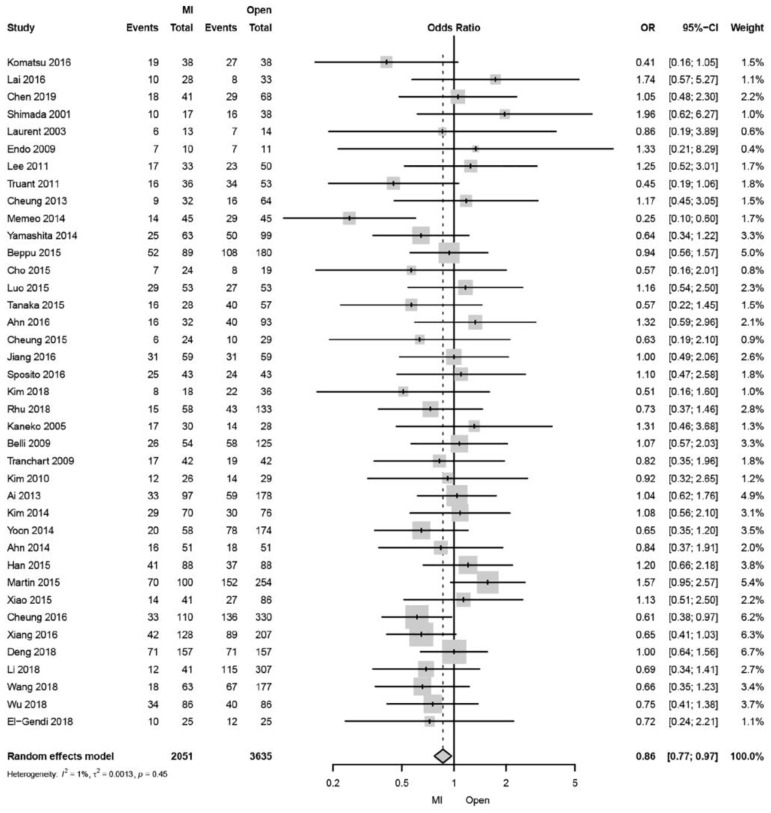
Forest plot of disease-specific 3-year mortality comparing minimally invasive and open liver resection for hepatocellular carcinoma. The studies shown in this figure can be found as references [6,21,23,25,26,27,28,29,30,31,32,33,34,35,36,37,38,39,40,41,42,43,44,45,46,47,48,49,50,51,52,53,54,55,56,57,58]. Reprinted/adapted with permission from Ref. [24]. 2021, SAGE Publications.

Hepatocellular carcinoma (HCC) is the most common primary liver tumor and the third leading cause of cancer-related death worldwide [1,2]. Liver resection is a valuable treatment modality in patients with HCC with preserved liver function [3,4]. The first laparoscopic liver resection (LLR) was reported in 1992, whereas the first LLR for HCC was in 1995 [5]. The LLR application was considered controversial for many years. Progress in laparoscopic techniques and expertise in combination with technological advances have led to more widespread adoption of minimally invasive approaches for HCC resection over the last 15 years [6]. Subsequently, the number of LLR cases increased due to the roadmap advocacy for the widespread use of safe LLR at numerous international consensus conferences [7,8,9,10] and the development of a difficulty scale classification [11,12,13,14]. Additionally, in Japan, the number and the proportion of LLR for the total number of liver resections increased from 1848 cases (9.9%) in 2011 to 5648 (24.8%) in 2017 [15]. At present, solitary lesions (≤5 cm) located in segments 2 through 6, which was the most acceptable LLR indication, as well as laparoscopic major liver resection, have been performed [7,8,14,16,17,18,19,20]. With these LLR developments, perioperative outcomes are better in patients with HCC who underwent LLR than those who underwent OLR, with no difference in long-term outcomes [16,17,21,22,23], whereas a recent systematic review and meta-analysis study indicated better long-term outcomes after minimal invasive liver resection (MILR), including LLR (48 articles) and robot-assisted liver resection (RALR, 2 articles) for HCC than OLR among the recently published data [24]. The pooled analysis revealed an 18% decrease in disease-specific 3-year mortality after MILR (almost, LLR) compared with OLR (Figure 1), and the sensitivity analysis of contemporary studies from 2010 to 2019 revealed a significantly lower 5-year all-cause mortality and 3-year disease-specific mortality in MILR compared to OLR. Thus, the overall picture is important in the surgical HCC treatment; however, factors such as cirrhosis due to background liver disease, repeat liver resection for HCC recurrence, advanced age, and obesity must be considered.

We reviewed the short- and long-term results of LLR usefulness (vs. OLR) with a special focus on these factors. Additionally, the usefulness of RALR, which has become increasingly popular in recent years, is discussed.

## 2. Liver Cirrhosis

**Figure 2:**
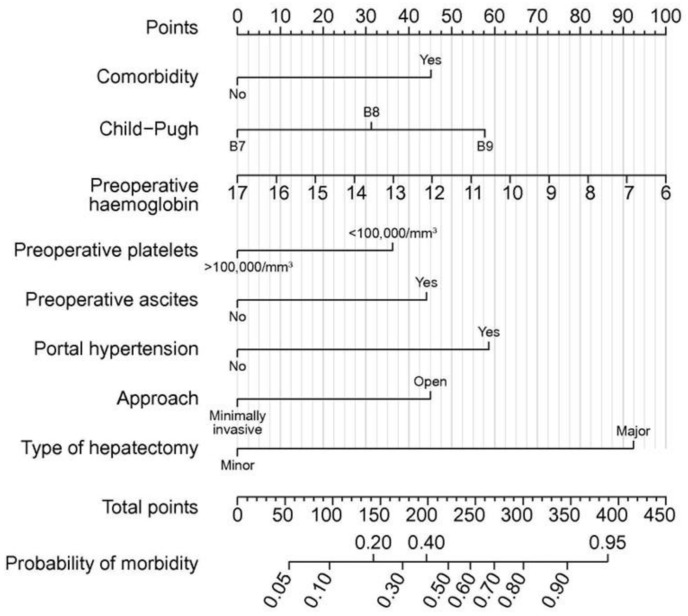
Nomogram for predicting 90-day morbidity after liver resection for hepatocellular carcinoma in patients with Child-Pugh class B. Nomogram was drawn using the multivariable logistic model for 90-day morbidity. Reprinted/adapted with permission from Ref. [70]. 2019, Elsevier.

Most patients with HCC commonly have chronic hepatitis and cirrhosis. Liver resection for patients with cirrhosis is challenging due to elevated portal pressure and impaired coagulation function. One systematic review and meta-analysis [59], one systematic review [60], and two meta-analyses [61,62] compared LLR with OLR for patients with cirrhosis with HCC. These reports revealed no difference in operation time among patients who underwent LLR and OLR; however, LLR reports decreased blood loss, transfusion rate, postoperative complications (including postoperative ascites and liver failure), and length of hospital stay. Moreover, LLR gains better 1-year overall survival (OS) [61,62] and 5-year OS [60,61,62]. Only one report revealed better 1-year disease-free survival (DFS) in LLR than in OLR [61]. However, among patients with cirrhosis, patients with Child-Pugh class B were reported to have more complications and deaths in the hospital and poorer long-term outcomes than patients with Child-Pugh class A [63,64,65,66,67], but the effect of LLR remains controversial because of the small number of patients [68,69]. Recently, Berardi et al. [70] reported an international multicenter study of 253 patients with Child-Pugh class B regarding short- and long-term outcomes. The comorbidity prevalence, increased Child-Pugh score (7 to 9), decreased preoperative hemoglobin and platelet count, and preoperative ascites and portal hypertension prevalence, increased the risk for postoperative complication within 90 days postoperatively (Figure 2). Moreover, minimally invasive surgery, including LLR and minor liver resection, decreased the risk for postoperative complications. Additionally, LLR did not affect DFS or OS rates. Liver cirrhosis is a well-known risk factor for postoperative liver failure-related mortality [71]. However, the development of devices, hemostasis techniques, and pneumoperitoneum and minimization of delamination in the LLR has controlled the bleeding and prevented postoperative ascites [25,72], which might lead to postoperative early recovery even for patients with Child-Pugh class B cirrhosis. Some better LLR prognoses might be caused by less compression during laparoscopic manipulation, which prevented tumor cell metastasis [62]. However, several reports revealed that LLR has no effect on long-term prognosis (no difference from OLR) [25,73], and only tumor factors were found to determine DFS in a study of Child-Pugh class B, while tumor factors and systemic status, including cirrhosis, determine OS [70]. LLR may be a useful treatment for patients who may not have previously been candidates for open surgery and may even prolong survival. However, further study is needed on the efficacy of LLR on long-term outcomes after cirrhotic liver resection.

## 3. Laparoscopic Repeat Liver Resection (LRLR) for Recurrent HCC

High recurrence even after curative liver resection for the initial HCC is a significant oncologic feature of HCC [74,75,76,77]. Additionally, hepatic resection is recommended for HCC recurrences (HCCR), as well as primary cases, if HCC has ≤3 nodules [4]. However, adhesions after initial hepatectomy are not only seen on the liver dissection surface, but also on the dissection surface and hepatoduodenal mesentery at a certain frequency, which makes repeat liver resection difficult, leading to unexpected blood loss and vascular or biliary structure intraoperative injury [78,79,80]. Conversely, some reported the remits of LRLR, such as minimalization of dissection of the adhesion under high magnification directly from the caudal direction [13,81] and small targeted area without damages to the surrounding area in the LRLR [79]. Some highly experienced centers reported feasible and safe LRLR for HCCR in the single-arm study [81,82,83,84]. A meta-analysis revealed that LRLR (*n* = 145) had a lower rate of in-hospital complication, much less blood loss, and a shorter hospital stay than open repeat liver resection (ORLR, *n* = 190) [85]. However, these studies were very small in number. Recently, an international collaborative study by Morise et al. [86] examined the usefulness of LRLR (*n* = 648) for HCCR and compared ORLR (*n* = 934) using propensity score matching (PSM, each, *n* = 238). The operation time was longer in the PSM cohort (mean, 273 min vs. 232 min, *p* = 0.007), but blood loss was lower (mean, 268 mL vs. 497 mL, *p* = 0.001) in patients who underwent LRLR than in those who did ORLR. No differences were found in the incidence of postoperative 90-day complications, 90-day mortality, length of hospital stay, or long-term survival. Therefore, case selection that would benefit from LRLR would be important. Kinoshita, et al. [87] reported the difficulty of LRLR in 60 patients with HCCR. Additionally, (1) an open approach during previous liver resection, (2) two or more previous liver resections, (3) a history of previous liver resection with not less than a sectionectomy, (4) a tumor near the resected site of the previous liver resection, and (5) intermediate or high difficulty in the difficulty scoring system [11] were independent risk factors for prolonged operative time and/or severe adhesion of LRLR. Thereafter, they validated less blood loss and lower postoperative complication incidence in LRLR than in ORLR among patients with ≤3 applicable risk factors; however, the operation time was longer in LRLR than in ORLR, and no difference was observed in other intra- and postoperative outcomes among LRLR and ORLR in patients with ≥4 of these 5 variables, suggesting that LRLR has no advantage in these patients [88]. On the basis of these findings, LRLR may have better short-term results than ORLR, but preoperative evaluation, such as details of prior surgeries, will be needed to determine whether it can be safely applied.

## 4. Elderly

The geriatric population has dramatically increased, and the number of elderly patients who undergo liver resection has even more rapidly increased [89]. Some reports revealed that the incidences of postoperative complication and mortality were comparable between elderly and non-elderly patients in OLR [90,91], but others have revealed an increased mortality incidence in elderly patients [92]. The reported incidence of overall postoperative complications in the elderly (aged 65–75 years) ranged from 29% to 59%, that of major complications (Clavien–Dindo grade ≥ IIIa) ranged from 16% to 41%, and that of mortality ranged from 0% to 9% [90,91,92,93]. Large-scale data from the Diagnosis Procedure Combination database, a national administrative database in Japan (2007–2012, *n* = 27,094), indicated the incidence of postoperative complication and mortality after liver resection increased up until the 70 s; however, no differences were found among patients aged in their 70 s, 80–84 years, and ≥85 years [94]. These results may be attributed to the fact that the adaptation is strictly handled for the elderly. Nomi et al. [95] reported a lower incidence of overall postoperative and major complications in elderly patients (aged ≥75 years) with HCC who underwent LLR than in those who underwent OLR, but others reported no difference among LLR and OLR [96,97]. However, LLR shortened the length of hospital stay [95,96,97]. One systematic review and meta-analysis using 12 studies (LLR: *n* = 831 and OLR; *n* = 931) indicated that LLR decreased the intraoperative blood loss, incidence of overall postoperative complications, including liver failure, ascites, and surgical site infection, major complication, and length of hospital stay although it includes all diseases, not just HCC [98]. Therefore, age would not be a determining factor for surgery. However, the high incidence of “elderly-related events”, including respiratory complications (pneumonia and respiratory failure requiring reintubation) [91], cardiac events [90], delirium [90,99], and discharge to rehabilitation facilities [99] are a major problem for liver resection in the elderly. LLR was reported to decrease the incidence of elderly-related events such as cardiopulmonary complications [95,96,100]. Moreover, maintenance of independence after liver resection is very important for elderly patients who underwent liver resection. Our previous study indicated that LLR decreased the incidence of postoperative loss of independence during the early postoperative period, including transfer to rehabilitation facilities, readmission within 30 days, discharge with any health care supports, and/or death within 90 days except cancer-related death, and at 1 year after liver resection, including the need of any healthcare supports and/or death due to deterioration of physical function [101,102]. A few studies reported regarding long-term survival; however, no differences were found in DFS or OS rate among elderly patients with HCC who underwent LLR or OLR [97]. LLR for the elderly has better intraoperative outcomes and fewer postoperative complications than OLR. In addition, LLR may have advantages to reduce elderly-related events and maintain independent living.

## 5. Obesity

**Figure 3:**
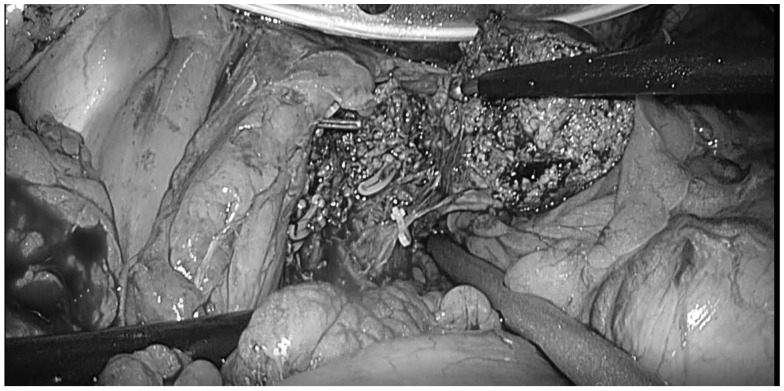
Laparoscopic liver resection for tumor located at segment I in a patient with obesity. Taking advantage of the Caudal approach of laparoscopic surgery, liver resection was performed with a good field of view despite the surgical depth.

The prevalence of obesity and its associated diseases has remained increasing worldwide. The prevalence of obesity (body mass index [BMI] of ≥30 kg/m^2^) is 40% in the United States [103] and approximately 20% in Europe [104]. In Japan, obesity is defined by a BMI of ≥25 kg/m^2^ [105]. As of 2018, 32.2% of males and 21.9% of females aged ≥20 years were classified as obese [106]. Furthermore, several reports revealed that patients with obesity are at high risk of developing HCC [107,108]. Thus, a higher prevalence of obesity and expansion of liver resection indications could increase the number of liver resections among patients with obesity with HCC in the future. Obesity is correlated with comorbidities and technical difficulties in open surgery and is considered a risk factor for postoperative complications in several surgical fields [109,110]. Countermeasures for the depth of the surgical field and large volume of intraperitoneal fat are important in abdominal surgery, including liver resection, in patients who are overweight and obese [111,112]. These situations are associated with increased operation time, blood loss, and postoperative complications in the OLR [113,114,115]. Liver parenchyma dissection and hepatic hilum treatment are sometimes challenging despite a large skin incision and gastrointestinal tract and greater momentum compression in OLR [116,117,118]. In contrast, pneumoperitoneum, head-up position, and high magnification—even at deep portions in the caudal view—can provide sufficient free space to control the forceps in LLR, even in patients who are overweight and obese (Caudal approach, Figure 3) [119,120,121]. There is some disagreement as to whether obesity increases the risk of conversion [12,111,113,122,123], but the LLR is reported to decrease intraoperative blood loss and postoperative complications compared with OLR even in obesity [113,118,121,124]. Moreover, obesity did not affect conversion rate, operation time, or blood loss in the LLR compared with non-obesity [113,122,123]. There is some disagreement regarding conversion to open surgery, but LLR has better short-term outcomes than OLR. Therefore, LLR for obesity would be feasible and safe.

## 6. Robot-Assisted Liver Resection (RALR)

RALRs are slowly spreading, although at a slower speed than LLRs [125,126]. In 2018, an international expert panel published a consensus guideline on the use of robotics in liver surgery, concluding that “RALR is as safe and feasible as LLR and OLR” for both major and minor liver resection [127]. Advantages of RALR include stability and magnification of a three-dimensional view, the best possible ergonomics, enhanced suturing capacity, the ability to complete more extensive or complex minimally invasive operations, integrated fluorescence guidance, and a shortened learning curve. However, the robotic platform remains limited by a paucity of parenchymal transection devices, a complete lack of hepatic feedback, and an additional operation time associated with docking and instrument exchange [128,129].

Some reported learning curves for LLR in 35 to 75 cases regarding operation time and incidence of liver injury (liver ischemia, congestion, or portal vein thrombosis) [130,131,132,133]. Conversely, early proponents of the robotic platform felt that robotic operations would be easier to learn than their laparoscopic counterparts due to the intentionally intuitive nature of robotic instrument us even for novice surgeons [134,135,136]. Some studies indicated shortened learning curves of 15 to 52 cases in RALR [137,138,139]. Additionally, the best possible ergonomics would increase the number of major hepatectomies and/or highly difficult cases [140,141,142]. However, RALR may become mainstream in the future. Some meta-analyses indicated less blood loss and a lower proportion of transfusion and incidence of postoperative complications in patients who underwent RALR than OLR [143,144,145]. Moreover, Kamarajah et al. [146] reported a systematic review and meta-analysis that included 26 articles and 2630 patients (RARL: 950 patients and LLR: 1680 patients) and revealed that blood loss was less (median, 286 mL vs. 301 mL, *p* < 0.001) and operation time was longer (median, 281 min vs. 221 min, *p* < 0.001) in patients who underwent RALR than in those who underwent LLR. Additionally, no difference was found in the incidence of postoperative complications, mortality, or length of hospital stay among patients who underwent RALR and LLR although readmission was lower in patients who underwent RALR than in those who underwent LLR. Moreover, a meta-analysis for major hepatectomy revealed an association between RALR and lower blood loss and conversion rate but with a slightly longer hospital stay compared to LLR [147]. Zhu et al. [148] revealed intra- and postoperative outcomes among patients who underwent RALR (*n* = 71), LLR (*n* = 141), and OLR (*n* = 157) for HCC; operation time was shortest and the length of hospital stay was longest in patients who underwent OLR, and similar results were demonstrated between those who did RALR and LLR. Conversely, some studies reported a higher incidence of postoperative bile leakage after RALR [149,150,151]. RALR is easy to manipulate in the hepatic hilum, but the lack of tactile sensation may cause inadvertent bile duct injury. In contrast, careful infraphrenic dissection was reported to reduce the incidence of postoperative pleural effusions [150]. Therefore, RALR does not significantly differ from LLR and is considered less invasive than OLR in terms of short-term results. Few studies reported on long-term outcomes after RALR; however, Zhu et al. [148] revealed no difference in DFS or OS among patients who underwent RALR, LLR, and OLR. Hence, RALR is as good as LLR as MIS. RALR may provide better perioperative results than LLR with further equipment development.

## 7. Conclusions

In conclusion, liver resection for HCC requires consideration of various situations, such as liver cirrhosis, repeat liver resection, obesity, and the elderly, but LLR overcomes these situations and has equal or better outcomes compared to OLR. In the future, RALR is expected to develop as an MIS alongside LLR.

## Data Availability

Data sharing is not applicable to this article as no datasets were generated or analyzed during the current study.

## References

[1] Ferlay J., Soerjomataram I., Dikshit R., Eser S., Mathers C., Rebelo M., Parkin D.M., Forman D., Bray F. (2015). Cancer incidence and mortality worldwide: Sources, methods and major patterns in GLOBOCAN 2012. Int. J. Cancer.

[2] Siegel R.L., Miller K.D., Jemal A. (2018). Cancer statistics, 2018. CA Cancer J. Clin..

[3] Kokudo N., Takemura N., Hasegawa K., Takayama T., Kubo S., Shimada M., Nagano H., Hatano E., Izumi N., Kaneko S. (2019). Clinical practice guidelines for hepatocellular carcinoma: The Japan Society of Hepatology 2017 (4th JSH-HCC guidelines) 2019 update. Hepatol. Res..

[4] European Association for the Study of the Liver (2018). EASL Clinical Practice Guidelines: Management of hepatocellular carcinoma. J. Hepatol..

[5] Hashizume M., Takenaka K., Yanaga K., Ohta M., Kajiyama K., Shirabe K., Itasaka H., Nishizaki T., Sugimachi K. (1995). Laparoscopic hepatic resection for hepatocellular carcinoma. Surg. Endosc..

[6] Laurent A. (2003). Laparoscopic Liver Resection for Subcapsular Hepatocellular Carcinoma Complicating Chronic Liver Disease. Arch. Surg..

[7] Buell J.F., Cherqui D., Geller D.A., O’Rourke N., Iannitti D., Dagher I., Koffron A.J., Thomas M., Gayet B., Han H.S. (2009). The international position on laparoscopic liver surgery: The Louisville Statement, 2008. Ann. Surg..

[8] Wakabayashi G., Cherqui D., Geller D.A., Buell J.F., Kaneko H., Han H.S., Asbun H., O’rourke N., Tanabe M., Koffron A.J. (2015). Recommendations for laparoscopic liver resection: A report from the second international consensus conference held in Morioka. Ann. Surg..

[9] Cheung T.T., Han H.-S., She W.H., Chen K.-H., Chow P., Yoong B.K., Lee K.F., Kubo S., Tang C.N., Wakabayashi G. (2017). The Asia Pacific Consensus Statement on Laparoscopic Liver Resection for Hepatocellular Carcinoma: A Report from the 7th Asia-Pacific Primary Liver Cancer Expert Meeting Held in Hong Kong. Liver Cancer.

[10] Abu Hilal M., Aldrighetti L., Dagher I., Edwin B., Troisi R.I., Alikhanov R., Aroori S., Belli G., Besselink M., Briceno J. (2018). The Southampton Consensus Guidelines for Laparoscopic Liver Surgery. Ann. Surg..

[11] Ban D., Tanabe M., Ito H., Otsuka Y., Nitta H., Abe Y., Hasegawa Y., Katagiri T., Takagi C., Itano O. (2014). A novel difficulty scoring system for laparoscopic liver resection. J. Hepato-Biliary-Pancreat. Sci..

[12] Hasegawa Y., Wakabayashi G., Nitta H., Takahara T., Katagiri H., Umemura A., Makabe K., Sasaki A. (2017). A novel model for prediction of pure laparoscopic liver resection surgical difficulty. Surg. Endosc..

[13] Wakabayashi G., Cherqui D., Geller D.A., Han H.-S., Kaneko H., Buell J.F. (2014). Laparoscopic hepatectomy is theoretically better than open hepatectomy: Preparing for the 2nd International Consensus Conference on Laparoscopic Liver Resection. J. Hepato-Biliary-Pancreat. Sci..

[14] Kawaguchi Y., Fuks D., Kokudo N., Gayet B. (2018). Difficulty of Laparoscopic Liver Resection. Ann. Surg..

[15] Ban D., Tanabe M., Kumamaru H., Nitta H., Otsuka Y., Miyata H., Kakeji Y., Kitagawa Y., Kaneko H., Wakabayashi G. (2020). Safe Dissemination of Laparoscopic Liver Resection in 27,146 Cases Between 2011 and 2017 From the National Clinical Database of Japan. Ann. Surg..

[16] Han H.-S., Shehta A., Ahn S., Yoon Y.-S., Cho J.Y., Choi Y. (2015). Laparoscopic versus open liver resection for hepatocellular carcinoma: Case-matched study with propensity score matching. J. Hepatol..

[17] Takahara T., Wakabayashi G., Beppu T., Aihara A., Hasegawa K., Gotohda N., Hatano E., Tanahashi Y., Mizuguchi T., Kamiyama T. (2015). Long-term and perioperative outcomes of laparoscopic versus open liver resection for hepatocellular carcinoma with propensity score matching: A multi-institutional Japanese study. J. Hepato-Biliary-Pancreat. Sci..

[18] Takahara T., Wakabayashi G., Konno H., Gotoh M., Yamaue H., Yanaga K., Fujimoto J., Kaneko H., Unno M., Endo I. (2016). Comparison of laparoscopic major hepatectomy with propensity score matched open cases from the National Clinical Database in Japan. J. Hepato-Biliary-Pancreat. Sci..

[19] Tanaka S., Kawaguchi Y., Kubo S., Kanazawa A., Takeda Y., Hirokawa F., Nitta H., Nakajima T., Kaizu T., Kaibori M. (2019). Validation of index-based IWATE criteria as an improved difficulty scoring system for laparoscopic liver resection. Surgery.

[20] Tanaka S., Kubo S., Kanazawa A., Takeda Y., Hirokawa F., Nitta H., Nakajima T., Kaizu T., Kaneko H., Wakabayashi G. (2017). Validation of a Difficulty Scoring System for Laparoscopic Liver Resection: A Multicenter Analysis by the Endoscopic Liver Surgery Study Group in Japan. J. Am. Coll. Surg..

[21] Li W., Han J., Xie G., Xiao Y., Sun K., Yuan K., Wu H. (2018). Laparoscopic versus open mesohepatectomy for patients with centrally located hepatocellular carcinoma: A propensity score matched analysis. Surg. Endosc..

[22] Meguro M., Mizuguchi T., Kawamoto M., Ota S., Ishii M., Nishidate T., Okita K., Kimura Y., Hirata K. (2015). Clinical comparison of laparoscopic and open liver resection after propensity matching selection. Surgery.

[23] Xiang L., Li J., Chen J., Wang X., Guo P., Fan Y., Zheng S. (2016). Prospective cohort study of laparoscopic and open hepatectomy for hepatocellular carcinoma. Br. J. Surg..

[24] Kamarajah S.K., Gujjuri R.R., Hilal M.A., Manas D.M., White S.A. (2021). Does minimally invasive liver resection improve long-term survival compared to open resection for hepatocellular carcinoma? A systematic review and meta-analysis. Scand. J. Surg..

[25] Tanaka S., Takemura S., Shinkawa H., Nishioka T., Hamano G., Kinoshita M., Ito T., Kubo S. (2015). Outcomes of Pure Laparoscopic versus Open Hepatic Resection for Hepatocellular Carcinoma in Cirrhotic Patients: A Case-Control Study with Propensity Score Matching. Eur. Surg. Res..

[26] Komatsu S., Brustia R., Goumard C., Perdigao F., Soubrane O., Scatton O. (2015). Laparoscopic versus open major hepatectomy for hepatocellular carcinoma: A matched pair analysis. Surg. Endosc..

[27] Lai C., Jin R.-A., Liang X., Cai X.-J. (2016). Comparison of laparoscopic hepatectomy, percutaneous radiofrequency ablation and open hepatectomy in the treatment of small hepatocellular carcinoma. J. Zhejiang Univ. B.

[28] Chen K., Pan Y., Wang Y.-F., Zheng X.-Y., Liang X., Yu H., Cai X.-J. (2019). Laparoscopic Right Hepatectomy for Hepatocellular Carcinoma: A Propensity Score Matching Analysis of Outcomes Compared with Conventional Open Surgery. J. Laparoendosc. Adv. Surg. Tech..

[29] Shimada M., Hashizume M., Maehara S., Tsujita E., Rikimaru T., Yamashita Y., Tanaka S., Adachi E., Sugimachi K. (2001). Laparoscopic hepatectomy for hepatocellular carcinoma. Surg. Endosc..

[30] Endo Y., Ohta M., Sasaki A., Kai S., Eguchi H., Iwaki K., Shibata K., Kitano S. (2009). A Comparative Study of the Long-term Outcomes After Laparoscopy-assisted and Open Left Lateral Hepatectomy for Hepatocellular Carcinoma. Surg. Laparosc. Endosc. Percutaneous Tech..

[31] Lee K.F., Chong C.N., Wong J., Cheung Y.S., Wong J., Lai P. (2011). Long-Term Results of Laparoscopic Hepatectomy Versus Open Hepatectomy for Hepatocellular Carcinoma: A Case-Matched Analysis. World J. Surg..

[32] Truant S., Bouras A.F., Hebbar M., Boleslawski E., Fromont G., Dharancy S., Leteurtre E., Zerbib P., Pruvot F.R. (2011). Laparoscopic resection vs. open liver resection for peripheral hepatocellular carcinoma in patients with chronic liver disease: A case-matched study. Surg. Endosc..

[33] Cheung T.T., Poon R.T.P., Yuen W.K., Chok K.S.H., Jenkins C.R., Chan S.C., Fan S.T., Lo C.M. (2013). Long-Term Survival Analysis of Pure Laparoscopic Versus Open Hepatectomy for Hepatocellular Carcinoma in Patients with Cirrhosis. Ann. Surg..

[34] Memeo R., De’Angelis N., Compagnon P., Salloum C., Cherqui D., Laurent A., Azoulay D. (2014). Laparoscopic vs. Open Liver Resection for Hepatocellular Carcinoma of Cirrhotic Liver: A Case–Control Study. World J. Surg..

[35] Yamashita Y.-I., Ikeda T., Kurihara T., Yoshida Y., Takeishi K., Itoh S., Harimoto N., Kawanaka H., Shirabe K., Maehara Y. (2014). Long-Term Favorable Surgical Results of Laparoscopic Hepatic Resection for Hepatocellular Carcinoma in Patients with Cirrhosis: A Single-Center Experience over a 10-Year Period. J. Am. Coll. Surg..

[36] Beppu T., Wakabayashi G., Hasegawa K., Gotohda N., Mizuguchi T., Takahashi Y., Hirokawa F., Taniai N., Watanabe M., Katou M. (2015). Long-term and perioperative outcomes of laparoscopic versus open liver resection for colorectal liver metastases with propensity score matching: A multi-institutional Japanese study. J. Hepato-Biliary-Pancreat. Sci..

[37] Cho J.Y., Han H.-S., Yoon Y.-S., Choi Y., Lee W. (2015). Outcomes of laparoscopic right posterior sectionectomy in patients with hepatocellular carcinoma in the era of laparoscopic surgery. Surgery.

[38] Luo L., Zou H., Yao Y., Huang X. (2015). Laparoscopic versus open hepatectomy for hepatocellular carcinoma: Short- and long-term outcomes comparison. Int. J. Clin. Exp. Med..

[39] Ahn S., Cho A., Kim E.K., Paik K.Y. (2016). Favorable Long-Term Oncologic Outcomes of Hepatocellular Carcinoma Following Laparoscopic Liver Resection. J. Laparoendosc. Adv. Surg. Tech..

[40] Cheung T.T., Poon R.T.P., Dai W.C., Chok K.S.H., Chan S.C., Lo C.M. (2015). Pure Laparoscopic Versus Open Left Lateral Sectionectomy for Hepatocellular Carcinoma: A Single-Center Experience. World J. Surg..

[41] Jiang X., Liu L., Zhang Q., Jiang Y., Huang J., Zhou H., Zeng L. (2016). Laparoscopic versus open hepatectomy for hepatocellular carcinoma: Long-term outcomes. J. BUON.

[42] Sposito C., Battiston C., Facciorusso A., Mazzola M., Muscarà C., Scotti M., Romito R., Mariani L., Mazzaferro V. (2016). Propensity score analysis of outcomes following laparoscopic or open liver resection for hepatocellular carcinoma. Br. J. Surg..

[43] Kim W.-J., Kim K.-H., Kim S.-H., Kang W.-H., Lee S.-G. (2018). Laparoscopic Versus Open Liver Resection for Centrally Located Hepatocellular Carcinoma in Patients with Cirrhosis: A Propensity Score-matching Analysis. Surg. Laparosc. Endosc. Percutaneous Tech..

[44] Rhu J., Kim S.J., Choi G.S., Kim J.M., Joh J.-W., Kwon C.H.D. (2018). Laparoscopic Versus Open Right Posterior Sectionectomy for Hepatocellular Carcinoma in a High-Volume Center: A Propensity Score Matched Analysis. World J. Surg..

[45] Kaneko H., Takagi S., Otsuka Y., Tsuchiya M., Tamura A., Katagiri T., Maeda T., Shiba T. (2005). Laparoscopic liver resection of hepatocellular carcinoma. Am. J. Surg..

[46] Belli G., Limongelli P., Fantini C., D’Agostino A., Cioffi L., Belli A., Russo G. (2009). Laparoscopic and open treatment of hepatocellular carcinoma in patients with cirrhosis. Br. J. Surg..

[47] Tranchart H., Di Giuro G., Lainas P., Roudie J., Agostini H., Franco D., Dagher I. (2009). Laparoscopic resection for hepatocellular carcinoma: A matched-pair comparative study. Surg. Endosc..

[48] Kim H.-H., Park E.K., Seoung J.S., Hur Y.H., Koh Y.S., Kim J.C., Cho C.K., Kim H.J. (2011). Liver resection for hepatocellular carcinoma: Case-matched analysis of laparoscopic versus open resection. J. Korean Surg. Soc..

[49] Ai J.-H., Li J.-W., Chen J., Bie P., Wang S.-G., Zheng S.-G. (2013). Feasibility and Safety of Laparoscopic Liver Resection for Hepatocellular Carcinoma with a Tumor Size of 5–10 cm. PLoS ONE.

[50] Kim S.-J., Jung H.-K., Lee D.-S., Yun S.-S., Kim H.-J. (2014). The comparison of oncologic and clinical outcomes of laparoscopic liver resection for hepatocellular carcinoma. Ann. Surg. Treat. Res..

[51] Yoon S.-Y., Kim K.-H., Jung D.-H., Yu A., Lee S.-G. (2014). Oncological and surgical results of laparoscopic versus open liver resection for HCC less than 5 cm: Case-matched analysis. Surg. Endosc..

[52] Ahn K.S., Kang K.J., Kim Y.H., Kim T.-S., Lim T.J. (2014). A Propensity Score-Matched Case-Control Comparative Study of Laparoscopic and Open Liver Resection for Hepatocellular Carcinoma. J. Laparoendosc. Adv. Surg. Tech..

[53] Martin R.C.G., Mbah N.A., Hill R.S., Kooby D., Weber S., Scoggins C.R., Maithel S.K. (2015). Laparoscopic Versus Open Hepatic Resection for Hepatocellular Carcinoma: Improvement in Outcomes and Similar Cost. World J. Surg..

[54] Xiao L., Xiang L.-J., Li J.-W., Chen J., Fan Y.-D., Zheng S.-G. (2015). Laparoscopic versus open liver resection for hepatocellular carcinoma in posterosuperior segments. Surg. Endosc..

[55] Deng Z.-C., Jiang W.-Z., Tang X.-D., Liu S.-H., Qin L., Qian H.-X. (2018). Laparoscopic hepatectomy versus open hepatectomy for hepatocellular carcinoma in 157 patients: A case controlled study with propensity score matching at two Chinese centres. Int. J. Surg..

[56] Wang W.-H., Kuo K.-K., Wang S.-N., Lee K.-T. (2018). Oncological and surgical result of hepatoma after robot surgery. Surg. Endosc..

[57] Wu X., Huang Z., Lau W.Y., Li W., Lin P., Zhang L., Chen Y. (2018). Perioperative and long-term outcomes of laparoscopic versus open liver resection for hepatocellular carcinoma with well-preserved liver function and cirrhotic background: A propensity score matching study. Surg. Endosc..

[58] El-Gendi A., El-Shafei M., El-Gendi S., Shawky A. (2018). Laparoscopic Versus Open Hepatic Resection for Solitary Hepatocellular Carcinoma Less Than 5 cm in Cirrhotic Patients: A Randomized Controlled Study. J. Laparoendosc. Adv. Surg. Tech..

[59] Twaij A. (2014). Laparoscopic vs open approach to resection of hepatocellular carcinoma in patients with known cirrhosis: Systematic review and meta-analysis. World J. Gastroenterol..

[60] Chen J., Bai T., Zhang Y., Xie Z.-B., Wang X.-B., Wu F.-X., Li L.-Q. (2015). The safety and efficacy of laparoscopic and open hepatectomy in hepatocellular carcinoma patients with liver cirrhosis: A systematic review. Int. J. Clin. Exp. Med..

[61] Goh E.L., Chidambaram S., Ma S. (2018). Laparoscopic vs open hepatectomy for hepatocellular carcinoma in patients with cirrhosis: A meta-analysis of the long-term survival outcomes. Int. J. Surg..

[62] Pan Y., Xia S., Cai J., Chen K., Cai X. (2021). Efficacy of Laparoscopic Hepatectomy versus Open Surgery for Hepatocellular Carcinoma With Cirrhosis: A Meta-analysis of Case-Matched Studies. Front. Oncol..

[63] Kusano T., Sasaki A., Kai S., Endo Y., Iwaki K., Shibata K., Ohta M., Kitano S. (2009). Predictors and prognostic significance of operative complications in patients with hepatocellular carcinoma who underwent hepatic resection. Eur. J. Surg. Oncol. (EJSO).

[64] Giuliante F., Ardito F., Pinna A.D., Sarno G., Giulini S.M., Ercolani G., Portolani N., Torzilli G., Donadon M., Aldrighetti L. (2012). Liver Resection for Hepatocellular Carcinoma ≤3 cm: Results of an Italian Multicenter Study on 588 Patients. J. Am. Coll. Surg..

[65] Kabir T., Syn N.L., Tan Z.Z., Tan H.-J., Yen C., Koh Y.-X., Kam J.H., Teo J.-Y., Lee S.-Y., Cheow P.-C. (2020). Predictors of post-operative complications after surgical resection of hepatocellular carcinoma and their prognostic effects on outcome and survival: A propensity-score matched and structural equation modelling study. Eur. J. Surg. Oncol. (EJSO).

[66] Koh Y.X., Tan H.J., Liew Y.X., Syn N., Teo J.Y., Lee S.Y., Goh B.K., Goh G.B., Chan C.Y. (2019). Liver Resection for Nonalcoholic Fatty Liver Disease-Associated Hepatocellular Carcinoma. J. Am. Coll. Surg..

[67] Chen Y.-S., Hsieh P.-M., Lin H.-Y., Hung C.-M., Lo G.-H., Hsu Y.-C., Lu I.-C., Lee C.-Y., Wu T.-C., Yeh J.-H. (2021). Surgical resection significantly promotes the overall survival of patients with hepatocellular carcinoma: A propensity score matching analysis. BMC Gastroenterol..

[68] Brytska N., Han H.-S., Shehta A., Yoon Y.-S., Cho J.Y., Choi Y. (2015). Laparoscopic liver resection for hepatitis B and C virus-related hepatocellular carcinoma in patients with Child B or C cirrhosis. HepatoBiliary Surg. Nutr..

[69] Cai X., Liang X., Yu T., Liang Y., Jing R., Jiang W., Li J., Ying H. (2015). Liver cirrhosis grading Child-Pugh class B: A Goliath to challenge in laparoscopic liver resection?—Prior experience and matched comparisons. HepatoBiliary Surg. Nutr..

[70] Berardi G., Morise Z., Sposito C., Igarashi K., Panetta V., Simonelli I., Kim S., Goh B.K., Kubo S., Tanaka S. (2019). Development of a nomogram to predict outcome after liver resection for hepatocellular carcinoma in Child-Pugh B cirrhosis. J. Hepatol..

[71] Kubo S., Tsukamoto T., Hirohashi K., Tanaka H., Shuto T., Takemura S., Yamamoto T., Uenishi T., Ogawa M., Kinoshita H. (2004). Correlation Between Preoperative Serum Concentration of Type IV Collagen 7s Domain and Hepatic Failure Following Resection of Hepatocellular Carcinoma. Ann. Surg..

[72] Kanazawa A., Tsukamoto T., Shimizu S., Kodai S., Yamazoe S., Yamamoto S., Kubo S. (2013). Impact of laparoscopic liver resection for hepatocellular carcinoma with F4-liver cirrhosis. Surg. Endosc..

[73] Cheung T.T., Dai W.C., Tsang S.H.Y., Chan A.C.Y., Chok K.S.H., Chan S.C., Lo C.M. (2016). Pure Laparoscopic Hepatectomy Versus Open Hepatectomy for Hepatocellular Carcinoma in 110 Patients with Liver Cirrhosis. Ann. Surg..

[74] Koda M., Tanaka S., Takemura S., Shinkawa H., Kinoshita M., Hamano G., Ito T., Kawada N., Shibata T., Kubo S. (2018). Long-Term Prognostic Factors after Hepatic Resection for Hepatitis C Virus-Related Hepatocellular Carcinoma, with a Special Reference to Viral Status. Liver Cancer.

[75] Tanaka S., Iimuro Y., Hirano T., Hai S., Suzumura K., Fujimoto J. (2015). Outcomes of Hepatic Resection for Large Hepatocellular Carcinoma: Special Reference to Postoperative Recurrence. Am. Surg..

[76] Tanaka S., Shinkawa H., Tamori A., Takemura S., Takahashi S., Amano R., Kimura K., Ohira G., Kawada N., Kubo S. (2020). Surgical outcomes for hepatocellular carcinoma detected after hepatitis C virus eradiation by direct-acting antivirals. J. Surg. Oncol..

[77] Tanaka S., Shinkawa H., Tamori A., Takemura S., Uchida-Kobayashi S., Amano R., Kimura K., Ohira G., Nishio K., Tauchi J. (2021). Postoperative direct-acting antiviral treatment after liver resection in patients with hepatitis C virus-related hepatocellular carcinoma. Hepatol. Res..

[78] Kinoshita M., Tanaka S., Kodai S., Takemura S., Shinkawa H., Ohira G., Nishio K., Tauchi J., Kanazawa A., Kubo S. (2023). Increasing incidence and severity of post-hepatectomy adhesion around the liver may be influenced by the hepatectomy-related operative procedures. Asian J. Surg..

[79] Morise Z. (2018). Status and perspective of laparoscopic repeat liver resection. World J. Hepatol..

[80] Szomstein S., Menzo E.L., Simpfendorfer C., Zundel N., Rosenthal R.J. (2006). Laparoscopic Lysis of Adhesions. World J. Surg..

[81] Hu M., Zhao G., Xu D., Liu R. (2010). Laparoscopic Repeat Resection of Recurrent Hepatocellular Carcinoma. World J. Surg..

[82] Belli G., Cioffi L., Fantini C., D’Agostino A., Russo G., Limongelli P., Belli A. (2009). Laparoscopic redo surgery for recurrent hepatocellular carcinoma in cirrhotic patients: Feasibility, safety, and results. Surg. Endosc..

[83] Goh B.K.P., Teo J., Chan C., Lee S., Cheow P., Chung A.Y.F. (2016). Laparoscopic repeat liver resection for recurrent hepatocellular carcinoma. ANZ J. Surg..

[84] Tsuchiya M., Otsuka Y., Maeda T., Ishii J., Tamura A., Kaneko H. (2012). Efficacy of Laparoscopic Surgery for Recurrent Hepatocellular Carcinoma. Hepatogastroenterology.

[85] Cai W., Liu Z., Xiao Y., Zhang W., Tang D., Cheng B., Li Q. (2019). Comparison of clinical outcomes of laparoscopic versus open surgery for recurrent hepatocellular carcinoma: A meta-analysis. Surg. Endosc..

[86] Morise Z., Aldrighetti L., Belli G., Ratti F., Belli A., Cherqui D., Tanabe M., Wakabayashi G., Cheung T.T., Lo C.M. (2020). Laparoscopic repeat liver resection for hepatocellular carcinoma: A multicentre propensity score-based study. Br. J. Surg..

[87] Kinoshita M., Kanazawa A., Kodai S., Shimizu S., Murata A., Nishio K., Hamano G., Shinkawa H., Tanaka S., Takemura S. (2019). Difficulty classifications of laparoscopic repeated liver resection in patients with recurrent hepatocellular carcinoma. Asian J. Endosc. Surg..

[88] Kinoshita M., Kanazawa A., Tanaka S., Takemura S., Amano R., Kimura K., Shinkawa H., Ohira G., Nishio K., Kubo S. (2021). Indications of Laparoscopic Repeat Liver Resection for Recurrent Hepatocellular Carcinoma. Ann. Gastroenterol. Surg..

[89] Nanashima A., Abo T., Nonaka T., Fukuoka H., Hidaka S., Takeshita H., Ichikawa T., Sawai T., Yasutake T., Nakao K. (2011). Prognosis of patients with hepatocellular carcinoma after hepatic resection: Are elderly patients suitable for surgery?. J. Surg. Oncol..

[90] Nozawa A., Kubo S., Takemura S., Sakata C., Urata Y., Nishioka T., Kinoshita M., Hamano G., Uenishi T., Suehiro S. (2014). Hepatic resection for hepatocellular carcinoma in super-elderly patients aged 80 years and older in the first decade of the 21st century. Surg. Today.

[91] Wang W.-L., Zhu Y., Cheng J.-W., Li M.-X., Xia J.-M., Hao J., Yu L., Lv Y., Wu Z., Wang B. (2014). Major hepatectomy is safe for hepatocellular carcinoma in elderly patients with cirrhosis. Eur. J. Gastroenterol. Hepatol..

[92] Cook E.J., Welsh F.K.S., Chandrakumaran K., John T.G., Rees M. (2012). Resection of colorectal liver metastases in the elderly: Does age matter?. Color. Dis..

[93] Kishida N., Hibi T., Itano O., Okabayashi K., Shinoda M., Kitago M., Abe Y., Yagi H., Kitagawa Y. (2015). Validation of Hepatectomy for Elderly Patients with Hepatocellular Carcinoma. Ann. Surg. Oncol..

[94] Okinaga H., Yasunaga H., Hasegawa K., Fushimi K., Kokudo N. (2017). Short-Term Outcomes following Hepatectomy in Elderly Patients with Hepatocellular Carcinoma: An Analysis of 10,805 Septuagenarians and 2,381 Octo- and Nonagenarians in Japan. Liver Cancer.

[95] Nomi T., Hirokawa F., Kaibori M., Ueno M., Tanaka S., Hokuto D., Noda T., Nakai T., Ikoma H., Iida H. (2019). Laparoscopic versus open liver resection for hepatocellular carcinoma in elderly patients: A multi-centre propensity score-based analysis. Surg. Endosc..

[96] Goh B.K.P., Chua D., Syn N., Teo J.-Y., Chan C.-Y., Lee S.-Y., Jeyaraj P.R., Cheow P.-C., Chow P.K.H., Ooi L.L.P.J. (2018). Perioperative Outcomes of Laparoscopic Minor Hepatectomy for Hepatocellular Carcinoma in the Elderly. World J. Surg..

[97] Kim J.M., Kim S., Rhu J., Choi G.-S., Kwon C.H.D., Joh J.-W. (2020). Elderly Hepatocellular Carcinoma Patients: Open or Laparoscopic Approach?. Cancers.

[98] Mohamedahmed A.Y.Y., Zaman S., Albendary M., Wright J., Abdalla H., Patel K., Mankotia R., Sillah A.K. (2021). Laparoscopic versus open hepatectomy for malignant liver tumours in the elderly: Systematic review and meta-analysis. Updat. Surg..

[99] Cho S.W., Steel J., Tsung A., Marsh J.W., Geller D.A., Gamblin T.C. (2010). Safety of Liver Resection in the Elderly: How Important Is Age?. Ann. Surg. Oncol..

[100] Tanaka S., Ueno M., Iida H., Kaibori M., Nomi T., Hirokawa F., Ikoma H., Nakai T., Eguchi H., Kubo S. (2018). Preoperative assessment of frailty predicts age-related events after hepatic resection: A prospective multicenter study. J. Hepato-Biliary-Pancreat. Sci..

[101] Tanaka S., Iida H., Ueno M., Hirokawa F., Nomi T., Nakai T., Kaibori M., Ikoma H., Eguchi H., Shinkawa H. (2019). Preoperative Risk Assessment for Loss of Independence Following Hepatic Resection in Elderly Patients. Ann. Surg..

[102] Tanaka S., Iida H., Ueno M., Hirokawa F., Yoshida H., Ishii H., Nomi T., Nakai T., Kaibori M., Ikoma H. (2022). Postoperative loss of independence 1 year after liver resection: Prospective multicentre study. Br. J. Surg..

[103] Hales C.M., Fryar C.D., Carroll M.D., Freedman D.S., Ogden C.L. (2018). Trends in Obesity and Severe Obesity Prevalence in US Youth and Adults by Sex and Age, 2007-2008 to 2015-2016. JAMA.

[104] World Health Organization Obesity and Overweight. https://www.who.int/news-room/fact-sheets/detail/obesity-and-overweight.

[105] McCurry J. (2007). Japan battles with obesity. Lancet.

[106] Ministry of Health, Labour and Welfare of Japan Report of national health and nutrition 2018. https://www.mhlw.go.jp/content/10900000/000688863.pdf.

[107] Berentzen T.L., Gamborg M., Holst C., Sørensen T.I., Baker J.L. (2014). Body mass index in childhood and adult risk of primary liver cancer. J. Hepatol..

[108] Larsson S.C., Wolk A. (2007). Overweight, obesity and risk of liver cancer: A meta-analysis of cohort studies. Br. J. Cancer.

[109] Mullen J.T., Davenport D.L., Hutter M.M., Hosokawa P.W., Henderson W.G., Khuri S.F., Moorman D.W. (2008). Impact of Body Mass Index on Perioperative Outcomes in Patients Undergoing Major Intra-abdominal Cancer Surgery. Ann. Surg. Oncol..

[110] Dindo D., Muller M.K., Weber M., Clavien P.-A. (2003). Obesity in general elective surgery. Lancet.

[111] Yu X., Yu H., Fang X. (2015). The impact of body mass index on short-term surgical outcomes after laparoscopic hepatectomy, a retrospective study. BMC Anesthesiol..

[112] WHO Expert Consultation (2004). Appropriate body-mass index for Asian populations and its implications for policy and intervention strategies. Lancet.

[113] Ishihara A., Tanaka S., Shinkawa H., Yoshida H., Takemura S., Amano R., Kimura K., Ohira G., Nishio K., Kubo S. (2021). Superiority of laparoscopic liver resection to open liver resection in obese individuals with hepatocellular carcinoma: A retrospective study. Ann. Gastroenterol. Surg..

[114] Cucchetti A., Cescon M., Ercolani G., Di Gioia P., Peri E., Pinna A.D. (2011). Safety of hepatic resection in overweight and obese patients with cirrhosis. Br. J. Surg..

[115] Tanaka S., Iimuro Y., Hirano T., Hai S., Suzumura K., Nakamura I., Kondo Y., Fujimoto J. (2013). Safety of hepatic resection for hepatocellular carcinoma in obese patients with cirrhosis. Surg. Today.

[116] Gedaly R., McHugh P.P., Johnston T.D., Jeon H., Ranjan D., Davenport D.L. (2009). Obesity, Diabetes, and Smoking are Important Determinants of Resource Utilization in Liver Resection: A Multicenter Analysis of 1029 Patients. Ann. Surg..

[117] Balzan S., Nagarajan G., Farges O., Galleano C.Z., Dokmak S., Paugam-Burtz C., Belghiti J. (2010). Safety of Liver Resections in Obese and Overweight Patients. World J. Surg..

[118] Uchida H., Iwashita Y., Saga K., Takayama H., Watanabe K., Endo Y., Yada K., Ohta M., Inomata M. (2016). Benefit of laparoscopic liver resection in high body mass index patients. World J. Gastroenterol..

[119] Soubrane O., Schwarz L., Cauchy F., Perotto L.O., Brustia R., Bernard D., Scatton O. (2015). A Conceptual Technique for Laparoscopic Right Hepatectomy Based on Facts and Oncologic Principles. Ann. Surg..

[120] Tomishige H., Morise Z., Kawabe N., Nagata H., Ohshima H., Kawase J., Arakawa S., Yoshida R., Isetani M. (2013). Caudal approach to pure laparoscopic posterior sectionectomy under the laparoscopy-specific view. World J. Gastrointest. Surg..

[121] Kwan B., Waters P.S., Keogh C., Cavallucci D.J., O’Rourke N., Bryant R.D. (2021). Body mass index and surgical outcomes in laparoscopic liver resections: A systematic review. ANZ J. Surg..

[122] Nomi T., Fuks D., Ferraz J.-M., Kawaguchi Y., Nakajima Y., Gayet B. (2015). Influence of body mass index on postoperative outcomes after laparoscopic liver resection. Surg. Endosc..

[123] Ome Y., Hashida K., Yokota M., Nagahisa Y., Okabe M., Kawamoto K. (2019). The safety and efficacy of laparoscopic hepatectomy in obese patients. Asian J. Surg..

[124] Toriguchi K., Hatano E., Sakurai T., Seo S., Taura K., Uemoto S. (2015). Laparoscopic Liver Resection in Obese Patients. World J. Surg..

[125] Giulianotti P.C. (2003). Robotics in General Surgery. Arch. Surg..

[126] Patriti A., Ceccarelli G., Bartoli A., Spaziani A., Lapalorcia L.M., Casciola L. (2009). Laparoscopic and robot-assisted one-stage resection of colorectal cancer with synchronous liver metastases: A pilot study. J. Hepato-Biliary-Pancreat. Surg..

[127] Liu R., Wakabayashi G., Kim H.-J., Choi G.-H., Yiengpruksawan A., Fong Y., He J., Boggi U., Troisi R.I., Efanov M. (2019). International consensus statement on robotic hepatectomy surgery in 2018. World J. Gastroenterol..

[128] Ayabe R.I., Azimuddin A., Cao H.S.T. (2022). Robot-assisted liver resection: The real benefit so far. Langenbeck’s Arch. Surg..

[129] Troisi R.I., Pegoraro F., Giglio M.C., Rompianesi G., Berardi G., Tomassini F., De Simone G., Aprea G., Montalti R., De Palma G.D. (2019). Robotic approach to the liver: Open surgery in a closed abdomen or laparoscopic surgery with technical constraints?. Surg. Oncol..

[130] Vigano L., Laurent A., Tayar C., Tomatis M., Ponti A., Cherqui D. (2009). The Learning Curve in Laparoscopic Liver Resection. Ann. Surg..

[131] Nomi T., Fuks D., Kawaguchi Y., Mal F., Nakajima Y., Gayet B. (2015). Learning curve for laparoscopic major hepatectomy. Br. J. Surg..

[132] Lee W., Woo J.-W., Lee J.-K., Park J.-H., Kim J.-Y., Kwag S.-J., Park T., Jeong S.-H., Ju Y.-T., Jeong E.-J. (2016). Comparison of Learning Curves for Major and Minor Laparoscopic Liver Resection. J. Laparoendosc. Adv. Surg. Tech..

[133] Navarro J.G., Kang I., Rho S.Y., Choi G.H., Han D.H., Kim K.S., Choi J.S. (2020). Major Laparoscopic Versus Open Resection for Hepatocellular Carcinoma: A Propensity Score-Matched Analysis Based on Surgeons’ Learning Curve. Ann. Surg. Oncol..

[134] Lanfranco A.R., Castellanos A.E., Desai J.P., Meyers W.C. (2004). Robotic Surgery. Ann. Surg..

[135] Moore L., Wilson M., Waine E., Masters R.S.W., McGrath J.S., Vine S.J. (2014). Robotic technology results in faster and more robust surgical skill acquisition than traditional laparoscopy. J. Robot. Surg..

[136] Stewart C.L., Fong A., Payyavula G., DiMaio S., Lafaro K., Tallmon K., Wren S., Sorger J., Fong Y. (2021). Study on augmented reality for robotic surgery bedside assistants. J. Robot. Surg..

[137] Chen P.-D., Wu C.-Y., Hu R.-H., Chen C.-N., Yuan R.-H., Liang J.-T., Lai H.-S., Wu Y.-M. (2017). Robotic major hepatectomy: Is there a learning curve?. Surgery.

[138] Efanov M., Alikhanov R., Tsvirkun V., Kazakov I., Melekhina O., Kim P., Vankovich A., Grendal K., Berelavichus S., Khatkov I. (2017). Comparative analysis of learning curve in complex robot-assisted and laparoscopic liver resection. HPB.

[139] Zhu P., Liao W., Ding Z.-Y., Chen L., Zhang W.-G., Zhang B.-X., Chen X.-P. (2018). Learning Curve in Robot-Assisted Laparoscopic Liver Resection. J. Gastrointest. Surg..

[140] Chong C.C., Fuks D., Lee K.-F., Zhao J.J., Choi G.H., Sucandy I., Chiow A.K.H., Marino M.V., Gastaca M., Wang X. (2022). Propensity Score–Matched Analysis Comparing Robotic and Laparoscopic Right and Extended Right Hepatectomy. JAMA Surg..

[141] Chong C.C.N., Lok H.T., Fung A.K.Y., Fong A.K.W., Cheung Y.S., Wong J., Lee K.F., Lai P.B.S. (2019). Robotic versus laparoscopic hepatectomy: Application of the difficulty scoring system. Surg. Endosc..

[142] Lorenz E., Arend J., Franz M., Rahimli M., Perrakis A., Negrini V., Gumbs A.A., Croner R.S. (2021). Robotic and laparoscopic liver resection—Comparative experiences at a high-volume German academic center. Langenbeck’s Arch. Surg..

[143] Jiang B., Yan X.-F., Zhang J.-H. (2018). Meta-analysis of laparoscopic versus open liver resection for hepatocellular carcinoma. Hepatol. Res..

[144] Machairas N., Papaconstantinou D., Tsilimigras D.I., Moris D., Prodromidou A., Paspala A., Spartalis E., Kostakis I.D. (2019). Comparison between robotic and open liver resection: A systematic review and meta-analysis of short-term outcomes. Updat. Surg..

[145] Wong D.J., Wong M.J., Choi G.H., Wu Y.M., Lai P.B., Goh B.K.P. (2018). Systematic review and meta-analysis of robotic versus open hepatectomy. ANZ J. Surg..

[146] Kamarajah S.K., Bundred J., Manas D., Jiao L.R., Abu Hilal M., White S.A. (2020). Robotic versus conventional laparoscopic liver resections: A systematic review and meta-analysis. Scand. J. Surg..

[147] Coletta D., Sandri G.B.L., Giuliani G., Guerra F. (2020). Robot-assisted versus conventional laparoscopic major hepatectomies: Systematic review with meta-analysis. Int. J. Med. Robot. Comput. Assist. Surg..

[148] Zhu P., Liao W., Zhang W.-G., Chen L., Shu C., Zhang Z.-W., Huang Z.-Y., Chen Y.-F., Lau W.Y., Zhang B.-X.M. (2022). A Prospective Study Using Propensity Score Matching to Compare Long-term Survival Outcomes After Robotic-assisted, Laparoscopic, or Open Liver Resection for Patients with BCLC Stage 0-A Hepatocellular Carcinoma. Ann. Surg..

[149] Lee K.-F., Chong C., Cheung S., Wong J., Fung A., Lok H.-T., Lo E., Lai P. (2020). Robotic versus open hemihepatectomy: A propensity score-matched study. Surg. Endosc..

[150] Magistri P., Tarantino G., Guidetti C., Assirati G., Olivieri T., Ballarin R., Coratti A., Di Benedetto F. (2017). Laparoscopic versus robotic surgery for hepatocellular carcinoma: The first 46 consecutive cases. J. Surg. Res..

[151] Schmelzle M., Feldbrügge L., Galindo S.A.O., Moosburner S., Kästner A., Krenzien F., Benzing C., Biebl M., Öllinger R., Malinka T. (2022). Robotic vs. laparoscopic liver surgery: A single-center analysis of 600 consecutive patients in 6 years. Surg. Endosc..

